# Conformational rearrangements of the C1 ring in KaiC measure the timing of assembly with KaiB

**DOI:** 10.1038/s41598-018-27131-8

**Published:** 2018-06-11

**Authors:** Atsushi Mukaiyama, Yoshihiko Furuike, Jun Abe, Shin-ichi Koda, Eiki Yamashita, Takao Kondo, Shuji Akiyama

**Affiliations:** 1 Research Center of Integrative Molecular Systems (CIMoS), Institute for Molecular Science, National Institute for Natural Sciences, 38 Nishigo-Naka, Myodaiji, Okazaki 444-8585 Japan; 2 Department of Functional Molecular Science, SOKENDAI (The Graduate University for Advanced Studies), 38 Nishigo-Naka, Myodaiji, Okazaki 444-8585 Japan; 3 Institute for Protein Research, Osaka University, 3-2 Yamada-oka, Suita, 565-0871 Japan; 4 Division of Biological Science, Graduate School of Science, Nagoya University, Furo-cho, Chikusa-ku, Nagoya 464-8602 Japan

**Keywords:** Molecular conformation, Deformation dynamics, Oscillators

## Abstract

KaiC, the core oscillator of the cyanobacterial circadian clock, is composed of an N-terminal C1 domain and a C-terminal C2 domain, and assembles into a double-ring hexamer upon ATP binding. Cyclic phosphorylation and dephosphorylation at Ser431 and Thr432 in the C2 domain proceed with a period of approximately 24 h in the presence of other clock proteins, KaiA and KaiB, but recent studies have revealed a crucial role for the C1 ring in determining the cycle period. In this study, we mapped dynamic structural changes of the C1 ring in solution using a combination of site-directed tryptophan mutagenesis and fluorescence spectroscopy. We found that the C1 ring undergoes a structural transition, coupled with ATPase activity and the phosphorylation state, while maintaining its hexameric ring structure. This transition triggered by ATP hydrolysis in the C1 ring in specific phosphorylation states is a necessary event for recruitment of KaiB, limiting the overall rate of slow complex formation. Our results provide structural and kinetic insights into the C1-ring rearrangements governing the slow dynamics of the cyanobacterial circadian clock.

## Introduction

Circadian clocks are endogenous time-measuring systems used by various organisms to adapt their physiological activities to daily alterations in the environment. Cyanobacteria are the simplest organisms known to exhibit a circadian rhythm^1^. Their clock oscillator is composed of three proteins, KaiA, KaiB, and KaiC^2^. In the presence of KaiA and KaiB, KaiC rhythmically alters its own ATPase activity^3^, auto-phosphorylation/auto-dephosphorylation activities^4^, and assembly state with other Kai proteins^5^ with a period of approximately 24 h (Kai oscillator). The period of these rhythmic phenomena is minimally dependent on the temperature; this property, termed temperature compensation, is common to circadian systems from multiple species. KaiC is the core element of the Kai oscillator and provides a practical means for studying the essence of the clock mechanism^6^.

KaiC is composed of tandemly duplicated domains, an N-terminal (C1) domain and a C-terminal (C2) domain, and it forms a double-ring hexamer upon binding of ATP^7^. Cyclic phosphorylation/dephosphorylation of two residues (S431 and T432) in the C2 domain proceeds as follows: S/pT → pS/pT → pS/T → S/T → S/pT (where ‘S’ represents S431, ‘pS’ represents phosphorylated S431, ‘T’ represents T432, and ‘pT’ represents phosphorylated T432, respectively)^8–10^. KaiA promotes the auto-phosphorylation of KaiC, whereas KaiB attenuates the effect of KaiA^11^. Starting from these findings, the C2-ring structure has been studied in relation to phosphorylation state using various physicochemical techniques. Small-angle x-ray scattering measurements have shown that KaiC expands and contracts its C2 ring in a phosphorylation-dependent manner to control the timing of intermolecular interactions^12^. In addition, NMR studies using phospho-mimicking KaiC mutants have revealed that the flexibility of the C2 ring depends on its phosphorylation state^13^.

Subsequent studies focused on the C1 ring. A recent crystallographic study by our group revealed that a structural change of the C1 ring upon slow ATP hydrolysis (~11 ATP d^−1^) is a determinant of the period of the Kai oscillator^14^. Other groups reported that the C1 ring binds KaiB^15,16^. Accordingly, a dynamical aspect linking the two functions is of great interest as a key step to transmit slowness from intra-molecular to inter-molecular scales.

We performed a dynamic structural analysis of the C1 ring in solution using fluorescence spectroscopy. Using a series of KaiC mutants harboring a fluorescence probe for the C1-ring structure, we obtained evidence that the structural transition of the C1 ring is coupled with ATPase activity and the phosphorylation state and is the origin of the basic timing cue for assembly with KaiB.

## Results

### Design of KaiC mutants

**Figure 1:**
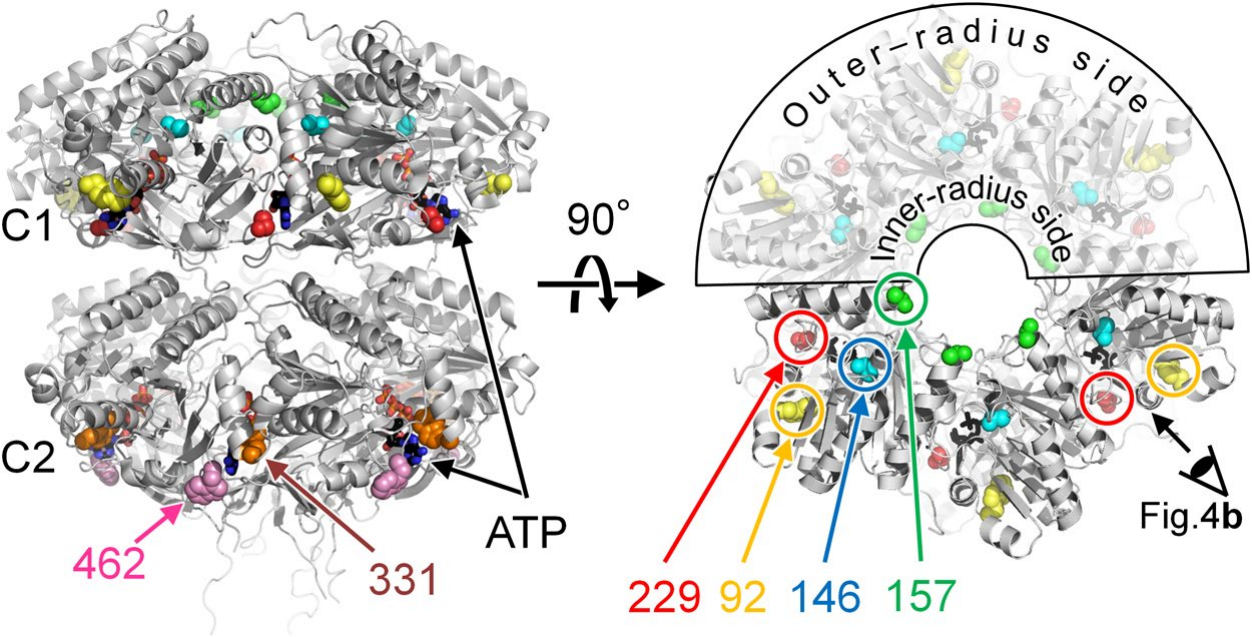
Design of KaiC mutants. Intrinsic (W92, W331, and W462) and designed (W146, W157, and W229) tryptophan residues mapped onto the crystal structure (accession code: 2GBL) of KaiC^7^.

KaiC intrinsically contains three Trp residues; one (W92) is located in the C1 domain, and the other two (W331 and W462) are in the C2 domain (Fig. 1). Initially, we tried to mask out the fluorescent contributions of these residues by replacing each tryptophan with phenylalanine. In contrast to the W92F substitution, however, both the W331F and W462F substitutions seriously impaired the robustness and/or circadian rhythmicity of the KaiC phosphorylation cycle (Supplementary Fig. S1). In addition, the expression levels of W331F/W462F, W92F/W462F, or W92F/W331F mutant in *E. coli* were much lower (<10%) than that of wild-type KaiC (KaiC^WT^). These results illustrate the practical difficulty of designing a tryptophan-free but functionally intact KaiC molecule.

Therefore, we updated our strategy by inserting a Trp residue as a probe into the KaiC^WT^ background, and then separating its fluorescence contribution from the other three residues. Substitutions were designed to cover potential structural changes of the C1 ring, as revealed in a previous study by our group^14^ (Fig. 1): S157W, to monitor fraying of the α7 helix on the inner-radius side of the hexamer; S146W, to detect the *cis*/*trans* peptide isomerization near the ATPase active site; and S229W, to monitor the protomer–protomer interface on the outer-radius side of the hexamer. KaiC^W92F^ was the exceptional Trp-masking mutant^12^ that enabled us to inspect the protein interior near W92 via comparison with KaiC^WT^. In the presence of KaiA and KaiB, both KaiC^S229W^ and KaiC^W92F^ were rhythmic with a prolonged period relative to that of KaiC^WT^, while stable oscillation was not observed for KaiC^S146W^ and KaiC^S157W^ (Supplementary Fig. S2).

### The C1 ring undergoes a conformational change in solution during auto-dephosphorylation process

**Figure 2:**
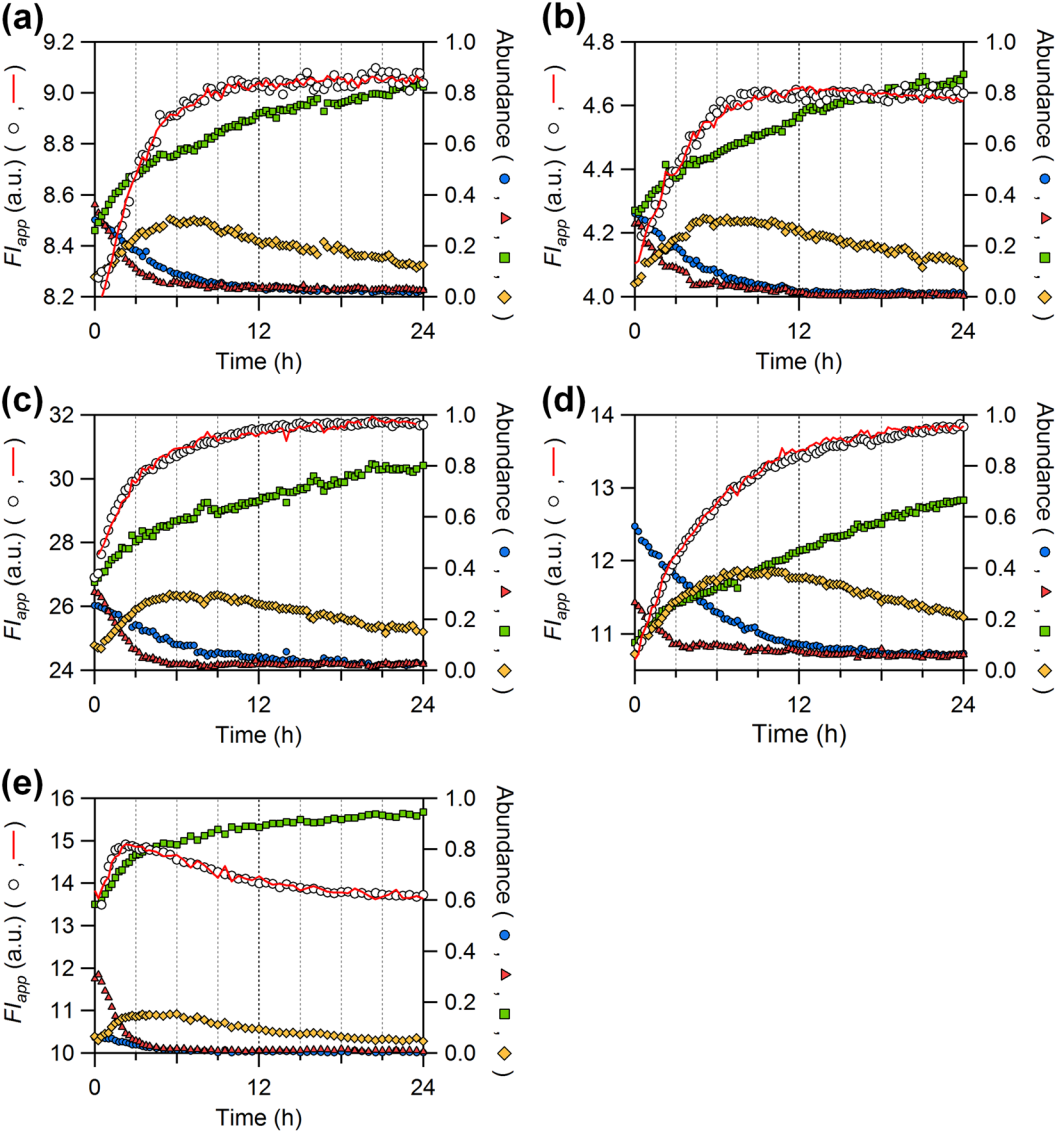
Time courses of tryptophan fluorescence intensity (*FI*) during KaiC auto-dephosphorylation. The apparent *FI* value (*FI*_app_, open circle) is compared with fractional abundances of the S/pT (red triangles), pS/pT (blue circles), pS/T (orange diamonds), and S/T (green squares) states of (**a**) KaiC^WT^, (**b**) KaiC^W92F^, (**c**) KaiC^S157W^, (**d**) KaiC^S229W^, and (**e**) KaiC^S146W^. In each panel, only one representative dataset from three independent experiments are shown for clarity of presentation. Slightly jagged appearance is seen in the resultant fits (red lines) of equation (1) to the data as a natural consequence of using the relative abundances (red triangles, blue circles, orange diamonds, green squares) pre-determined experimentally (see details in text and methods).

Figure 2 shows the time course of Trp fluorescence intensity (*FI*) integrated from 320 to 370 nm during auto-dephosphorylation of KaiC. As reported previously^12^, the apparent *FI* value (*FI*_app_, white circles in Fig. 2a) of KaiC^WT^ alone increased concomitantly with changes in the relative phosphorylation-state abundances (blue circles, red triangles, green squares, and orange diamonds in Fig. 2a) determined by densitmetric analysis of corresponding bands separated on SDS-PAGE gels^17^. Consistent with our previous observation^12^, the W92F substitution had minimum impact on the amplitude of the *FI*_app_ time course (Fig. 2b). On the other hand, the amplitudes of the *FI*_app_ increase of KaiC^S157W^ and KaiC^S229W^ were approximately 5- and 4-fold larger than that of KaiC^WT^, respectively, but exhibited somewhat similar temporal patterns (Fig. 2c,d). For KaiC^S146W^, the *FI*_app_ value exhibited a sharp increase during the first 2 h, followed by a gradual decrease (Fig. 2e).

**Figure 3:**
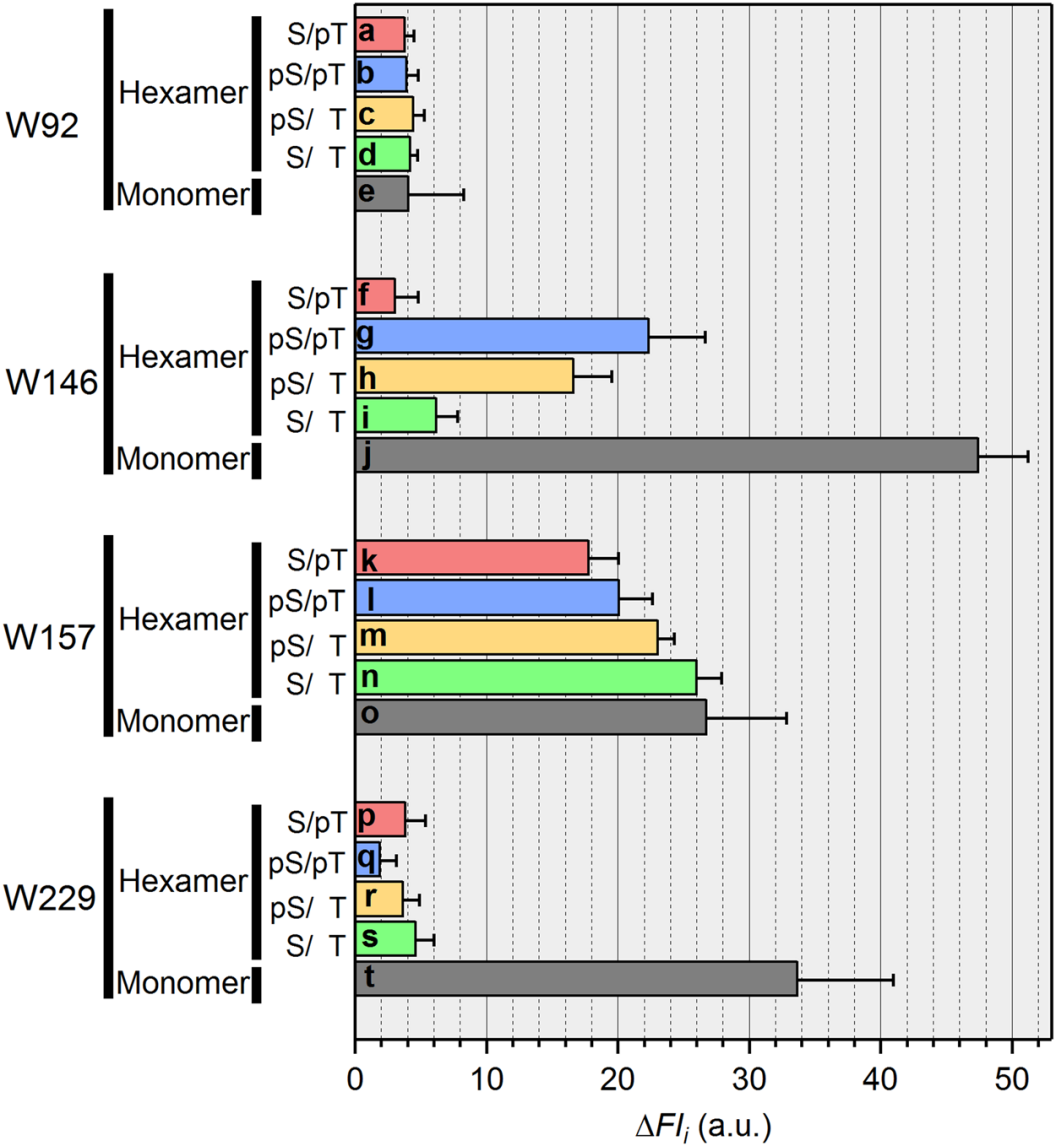
Fluorescence contributions of Trp residues probing the C1-ring structure of KaiC. Each contribution was estimated as the difference (Δ*FI*_*i*_, *i* = S/pT, pS/pT, pS/T, S/T) between KaiC^WT^ and the corresponding KaiC mutant; i.e., bar *a* in Fig. 3 corresponds to the subtraction of bar 6 from bar 1 in Fig. S3. Values shown in bars *a*–*t* are means ± S.E. from three independent experiments.

Using these data, we estimated the *FI* value of each phosphorylation state as described previously^12^. In brief, *FI*_app_(*t*) is assumed as a linear summation of the contributions from four phosphorylation states,

1 $$F{I}_{{\rm{app}}}(t)=F{I}_{{\rm{S}}/{\rm{pT}}}{A}_{{\rm{S}}/{\rm{pT}}}(t)+F{I}_{{\rm{pS}}/{\rm{pT}}}{A}_{{\rm{pS}}/{\rm{pT}}}(t)+F{I}_{{\rm{pS}}/{\rm{T}}}{A}_{{\rm{pS}}/{\rm{T}}}(t)+F{I}_{{\rm{S}}/{\rm{T}}}{A}_{{\rm{S}}/{\rm{T}}}(t)$$

where *A*_i_(*t*) is the relative phosphorylation-state abundance (*i* = S/pT, pS/pT, pS/T, S/T) pre-determined experimentally (blue circles, red triangles, green squares, and orange diamonds in Fig. 2). The *FI*_*i*_ values were then estimated by fitting equation (1) to *FI*_app_(*t*) (see details in Methods). Slightly jagged appearance was seen in the resultant fits (red lines in Fig. 2) as a natural consequence of using the relative abundances pre-determined experimentally. Next, we estimated the fluorescence contributions of each Trp probe as the *FI*_*i*_ difference (Δ*FI*_*i*_) between KaiC^WT^ and the Trp-inserted/-masked KaiC mutant (Fig. 3 and Supplementary Fig. S3). Although the fluorescence emission from W92 (bars *a*–*d*, Fig. 3) was almost insensitive to phosphorylation state, the emissions of W146 (bars *f*–*i*, Fig. 3) and W157 (bars *k*–*n*, Fig. 3) were changed depending on the phosphorylation state. The state-dependent variation of W146 and W157 clearly indicates that the C1 ring undergoes a structural change in solution.

**Figure 4:**
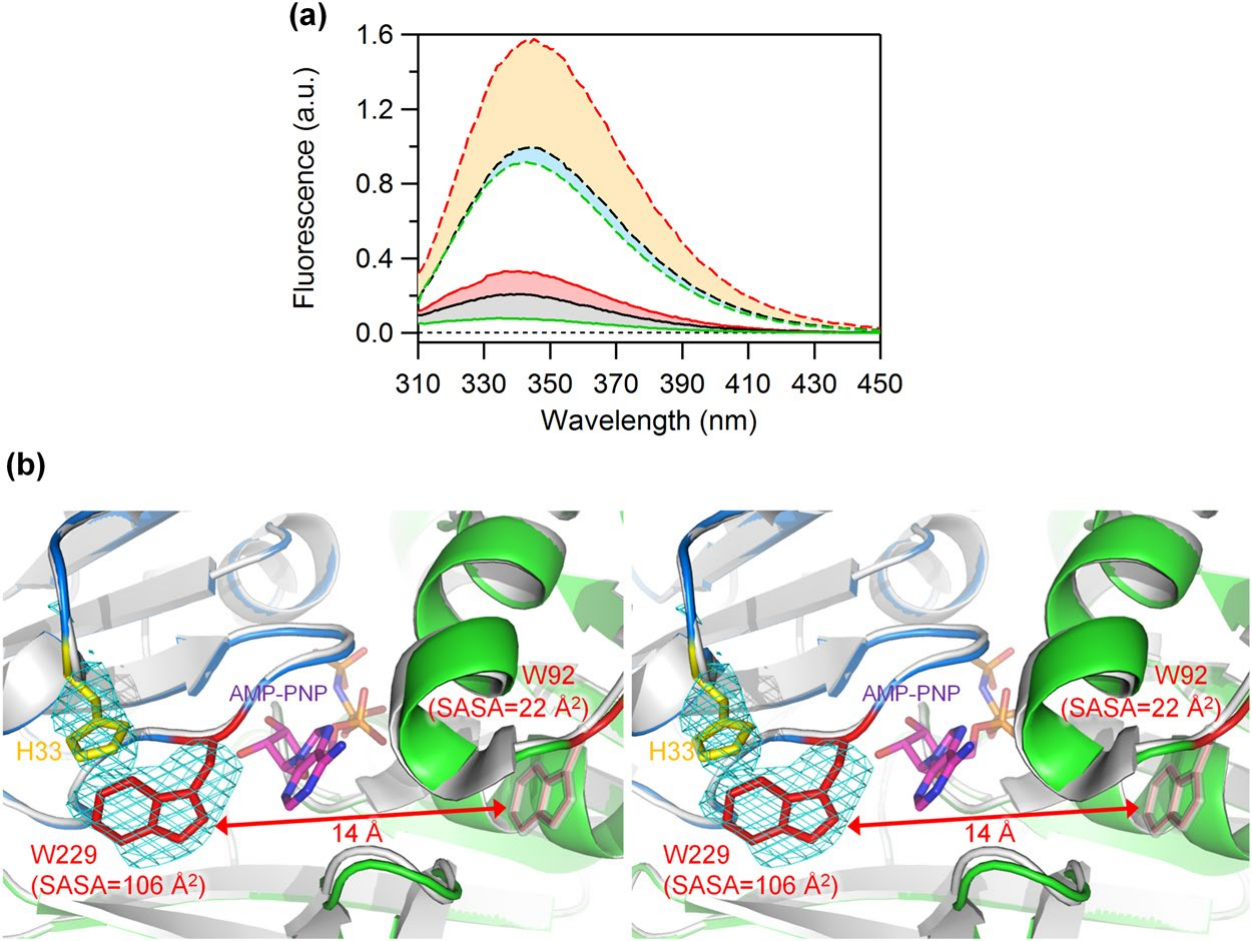
Fluorescence characterization of the global change of the C1 ring. (**a**) Trp-fluorescence spectra of KaiC^WT^ (black), KaiC^W92F^ (green), and KaiC^S229W^ (red) in the hexameric (solid lines) and monomeric forms (broken lines). Red and orange areas correspond to W229 fluorescence in the hexameric and monomeric forms, respectively. Gray and cyan regions correspond to W92 fluorescence in the hexameric and monomeric forms, respectively. (**b**) Zoomed-in stereo view of the C1–protomer interface of KaiC1^S229W^ (blue and green subunits, accession code: 5YZ8) superimposed onto KaiC1^WT^ (white subunits, accession code: 4TL7). W92 and W229 in KaiC1^S229W^ are drawn in red, and W92 in KaiC1^WT^ is drawn in white. The meshes represent the |*F*_o_| − |*F*_c_| difference Fourier maps for omitted side chains of H33 and W229 in KaiC1^S229W^ contoured at 2σ.

Minor differences in the emission of W229 among the four phosphorylation states (bars *p*–*s*, Fig. 3) suggest that the conformations around the protomer interface on the outer-radius side are slightly altered. To inspect the change in the global shape of the hexameric ring, we prepared quasi-monodispersed monomeric forms of KaiC and its mutants^18^, and then measured their fluorescence emissions as a reference (Fig. 4a and Supplementary Fig. S4). Emissions of the monomeric forms were dramatically more intense than those of the hexameric forms (Fig. 4a). W229 fluorescence in monomeric form (bar *t*, Fig. 3), which can be estimated from the difference between monomeric KaiC^WT^ and monomeric KaiC^S229W^ (orange area in Fig. 4a), was approximately 6-fold greater than that in hexameric KaiC^S229W^ (bars *p*–*s*, Fig. 3). By contrast, W92 fluorescence in monomeric form (bar *e*, Fig. 3) was comparable to that in hexameric form (bars *a*–*d*, Fig. 3). These contrasting results imply that W229 fluorescence is significantly quenched specifically in the hexameric form, whereas W92 fluorescence is quenched irrespective of the oligomerization state. This interpretation was further supported by the crystal structure of a C1-domain truncation, KaiC1^S229W^. The backbone RMSD value between KaiC1^S229W^ and KaiC1^WT^ was 0.38 Å (Fig. 4b), indicating that the mutation had minimal impact on the overall structure of the C1 ring. W92 is located in the interior of the C1 domain, and its solvent-accessible surface area (SASA) is quite small (22 Å^2^) in both the monomer and hexamer. In sharp contrast, because W229 is located near the C1–protomer interface of the hexamer, the SASA of W229 is buried by 36 Å^2^ following the monomer-to-hexamer transition (142 to 106 Å^2^). In fact (Fig. 4b), one edge of the indole ring of W229 is in close proximity to potential quenchers, including the adenine ring of bound ATP^19^ and a main-chain oxygen atom of I239^20^, and the other edge is nearly in contact with H33^21^. Collectively, these results indicate that the C1 ring undergoes a structural change while maintaining its hexameric structure.

### Fluorescence detection of the C1-ring deformation during assembly with KaiB

**Figure 5:**
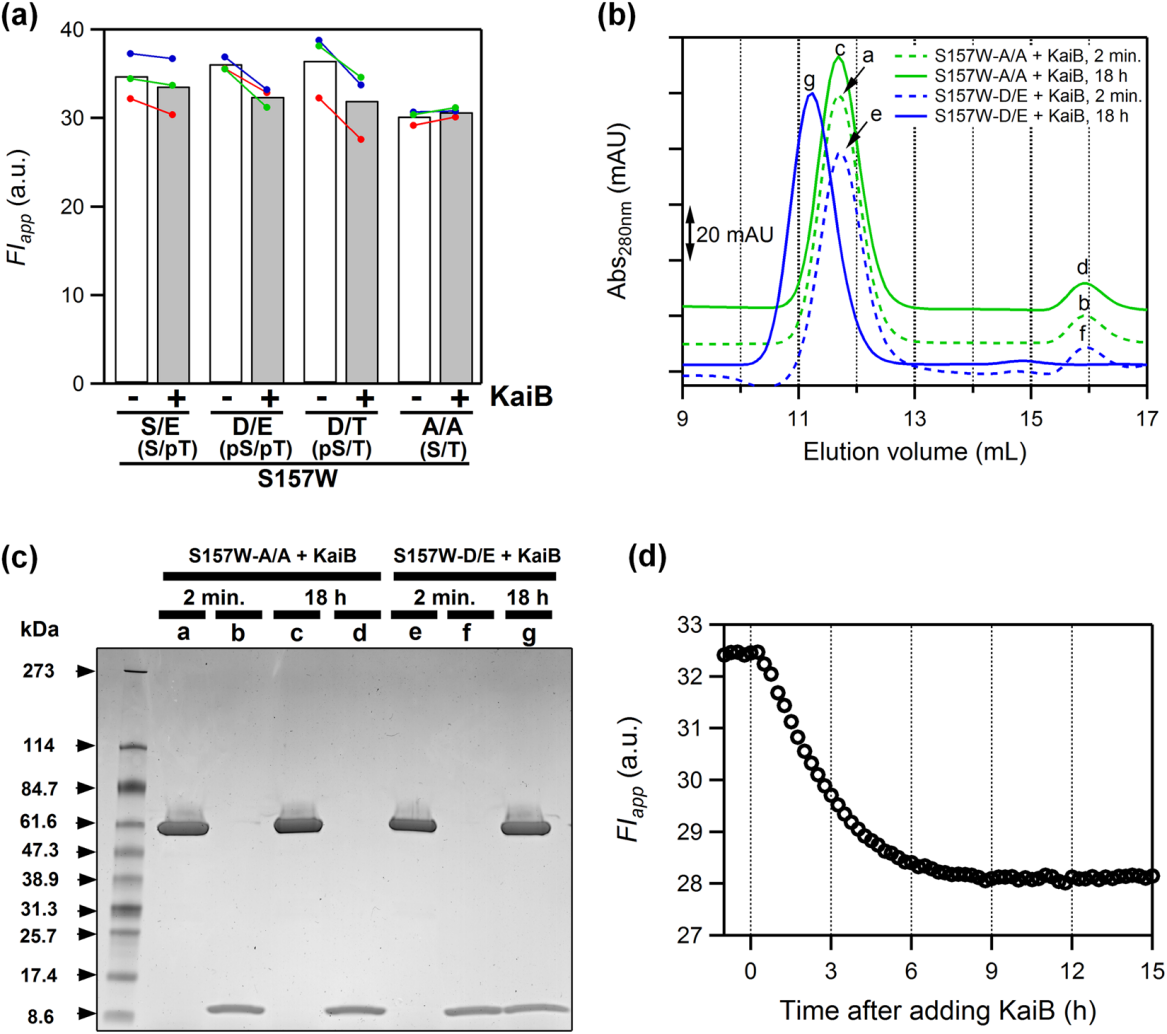
Fluorescence detection of the C1-ring rearrangements in the presence of KaiB. (**a**) Apparent fluorescence intensity (*FI*_*app*_) of the phospho-mimicking mutants, S/E, D/E, D/T and A/A of KaiC^S157W^ after 24 h incubation at 30 °C with or without an equimolar concentration of KaiB (monomer basis). Each dataset is shown in a different color as a pair of filled circles connected with a line, and the mean value from independent measurements is represented as bars. (**b**) Gel-filtration chromatograms of mixtures containing KaiB and KaiC^S157W^-A/A (green) or KaiC^S157W^-D/E (blue). The samples were loaded onto a column immediately after the mixing (dashed lines, *t* = 2 min) or after 18 h incubation at 30 °C (solid lines, *t* = 18 h). Each trace was vertically shifted for clarity of presentation. (**c**) SDS-PAGE analysis of peaks *a*–*g* in (**b**). Raw image is shown in Supplementary Fig S6. (**d**) Time course of *FI*_app_ for KaiC^S157W^-D/E before and after addition of an equimolar concentration of KaiB. The displayed trace corresponds to a green-colored dataset of KaiC^S157W^-DE in (**a**). A time of addition of KaiB was set as *t* = 0 h.

We found that W157 fluorescence is sensitive not only to the conformational change of the C1 ring (bars *k*–*n*, Fig. 3), but also to KaiB binding that is known to occur in the C1 ring^15,16^. We constructed a series of phospho-mimicking mutants of KaiC^S157W^ by introducing S431D (pS/T mimic), S431D/T432E (pS/pT mimic), S431A/T432A (S/T mimic), or T432E (S/pT mimic), and measured fluorescence spectra of these proteins after 24 h incubation at 30 °C with or without an equimolar concentration of KaiB (monomer basis). The co-incubation with KaiB resulted in quenching selectively for KaiC^S157W^-D/E and KaiC^S157W^-D/T (Fig. 5a). By contrast, the *FI* values of any phospho-mimicking mutants of KaiC^WT^ (no artificial Trp probes) were unaffected by the addition of KaiB (Supplementary Fig. S5). Because wild-type KaiB lacks Trp residues, these observations illustrate that W157 can be used to monitor the conformational change of the C1 ring during complex formation with KaiB. Gel-filtration chromatography analysis, revealing KaiB binding with KaiC^S157W^-D/E but not with KaiC^S157W^-A/A (Fig. 5b,c), further supports our interpretation that fluorescence quenching of KaiC^S157W^-D/E (Fig. 5a) reflects KaiB binding on the C1 ring. Consistently to the previous works^22–24^, these results demonstrate that the binding affinity of KaiB to the C1 ring is dependent on the phosphorylation state.

We also studied the kinetics of the structural change of the C1 ring upon addition of KaiB. For this purpose, pre-incubated KaiC^S157W^-D/E was mixed with KaiB, and then the mixture was subjected to time-resolved fluorescence measurement. Figure 5d shows the time course of the *FI*_app_ value of KaiC^S157W^-D/E after addition of an equimolar concentration of KaiB. Without any detectable burst phase, the *FI*_app_ value decreased slowly with *t*_1/2_ of 2.4 ± 0.04 h (*n* = 3), indicating slow complex formation with KaiB, as reported previously^5,25–27^.

### KaiB exclusively selects a post-hydrolysis conformation of the C1 ring for specific binding

**Figure 6:**
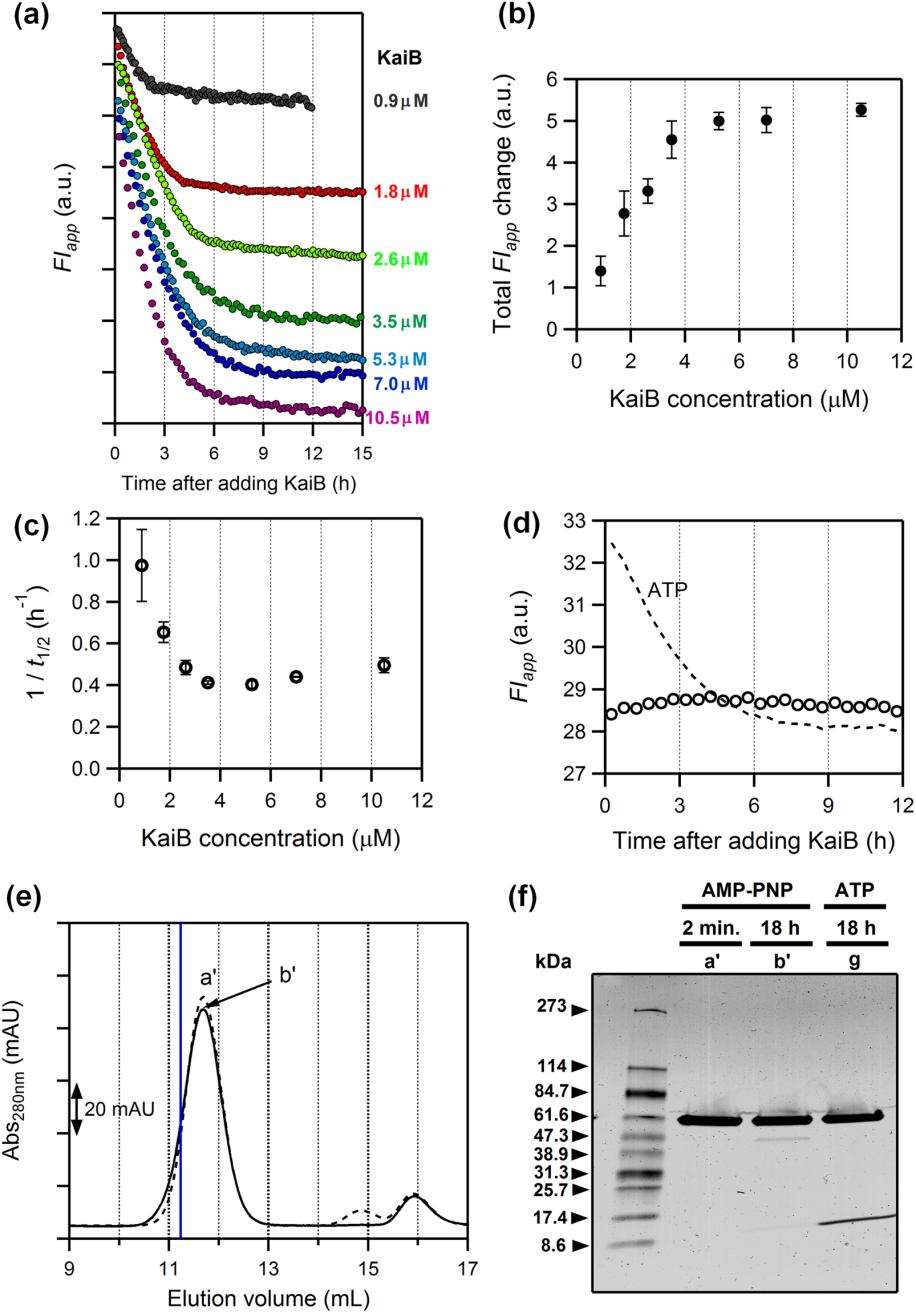
The ATP hydrolysis-coupled rearrangements of the C1 ring occurs before KaiB binding. (**a**) Time courses of apparent fluorescence intensity (*FI*_app_) of KaiC^S157W^-D/E in the presence of various concentrations of KaiB. Each plot was vertically shifted for clarity of presentation. (**b**) and (**c**) KaiB concentration dependences of total *FI*_app_ change (**b**) and inverse of the half-life time (1/*t*_1/2_) (**c**). The total *FI*_app_ change was determined as the difference in *FI*_app_ between the first and last time points. Values are means ± S.E. from three or more independent experiments. (**d**) Time course of *FI*_app_ for KaiC^S157W^-D/E upon addition of KaiB in the presence of 1 mM AMP-PNP (open circle) instead of 1 mM ATP (dashed line). (**e**) Gel-filtration chromatograms of mixtures containing KaiC^S157W^-D/E and KaiB in the presence of AMP-PNP. Samples were prepared by mixing KaiB and KaiC^S157W^-D/E, the latter of which was pre-incubated at 30 °C for 2 h after exchange of ATP with AMP-PNP. The mixtures were loaded onto a column immediately (dashed line) or after 18-h incubation at 30 °C (solid line). A blue line indicates the elution volume of the peak *g* in Fig. 5b. (**f**) SDS-PAGE analysis of peaks *a’* and *b’* in (**e**) and *g* in Fig. 5b. Raw image is shown in Supplementary Fig. S6.

The above results showed that KaiC alters the C1 ring conformation during complex formation, but it remained unclear whether the conformational change of the C1 ring occurs before binding with KaiB or is induced by KaiB-binding. To address this question, the dependence of the relaxation kinetics on KaiB concentration was examined by mixing a fixed concentration of KaiC^S157W^-D/E (3.5 µM) with 0.9–10.5 µM KaiB (Fig. 6a). Both total *FI*_app_ change between the initial and final time points (Fig. 6b) and the inverse of half-life time (1/*t*_1/2_) (Fig. 6c) revealed unique dependencies on the KaiB concentration. The total *FI*_app_ change was enhanced as KaiB concentration increased, and then became saturated at a KaiB concentration around 3.5 µM. This observation indicates that the total decrease in the *FI*_app_ value reflects the amount of KaiB bound to the C1 ring. Moreover, the plot of 1/*t*_1/2_ that decreased hyperbolically with increasing the KaiB concentration (Fig. 6c) is reminiscent of a conformational selection mechanism in which the conformational change in the protein occurs before ligand binding (Supplementary Fig. S7)^28^. Hence, our result suggests that KaiC changes the C1-ring conformation in order to bind with KaiB.

Finally, to investigate origins of the conformational selection of the C1 ring prior to KaiB binding, we investigated the effect of KaiC ATPase on the kinetics. We found that the value of 1/*t*_1/2_ at a KaiB concentration of 10.5 µM (~0.5 h^−1^) (Fig. 6c) was close to the measured ATPase activity of KaiC^S157W^-D/E in the absence of KaiB (12 ± 1 d^−1^ [*n* = 3]). Furthermore, exchanging external ATP with its non-hydrolyzable analog, adenylyl-imidophosphate (AMP-PNP), resulted in disappearance of the time-dependent change in *FI*_app_ after addition of KaiB (Fig. 6d). Under such solution condition, stable KaiB–KaiC complex was not detected even after long-term incubation (Fig. 6e,f). At the same time, it must be noted that inability of KaiC^S157W^-A/A to recruit KaiB (Fig. 5b,c) is not due to a deficiency of ATPase activity (31 ± 2 d^−1^ [*n* = 4]). Taken together, the complex formation between the C1 ring and KaiB is regulated both by the phosphorylation state (Fig. 5a) and by ATP hydrolysis-driven conformational change of the C1 ring (Fig. 6) to effect a rate-limiting step of the complex formation.

## Discussion

Tryptophan fluorescence is often used in studies of protein folding^29^, enzyme kinetics^30^, and protein–substrate interactions^21^ because of its high sensitivity to the local environment of the protein structure. In this study, we investigated the conformational change of the C1 ring using a series of Trp fluorescent probes incorporated into the C1 domain, enabling us to detect changes in the emission of W146 (bars *f*–*i*, Fig. 3) and W157 (bars *k*–*n*, Fig. 3). These changes were dependent on the phosphorylation state, clearly indicating that the C1 ring undergoes a structural transition in solution.

Fluorescence of Trp residues situated in or near protein–protein interfaces is often quenched^31–33^ or dequenched^34,35^ upon assembly or disassembly of protein molecules. Consistent with this, fluorescence of W229, near the C1–protomer interface, was dramatically quenched following the monomer-to-hexamer transition (bars *p*–*t*, Fig. 3). Thus, W229 fluorescence serves as a sensitive probe for C1-ring formation. By contrast, the phosphorylation state–dependent variation of W229 fluorescence (bars *p*–*s*, Fig. 3) was negligibly small. Therefore, the KaiC hexamer undergoes structural arrangement while maintaining the framework of the hexameric C1 ring.

It is worth discussing the state-dependent variation of the emission of W146 and W157 in terms of the structure transition of the C1 ring upon ATP hydrolysis. W146 fluorescence was dramatically dequenched during the transition from the S/pT to pS/pT states, and then quenched during the transition to the S/T state via the pS/T state. The radical increase and decrease in Δ*FI* indicate a substantial rearrangement of W146 during auto-dephosphorylation. Although the interpretation of W146 fluorescence is not straightforward, the drastic change in Δ*FI* is reminiscent of *cis*-to-*trans* isomerization of the D^145^S^146^ peptide upon ATP hydrolysis in the C1 domain^14^.

S157 controls N-terminal fraying of the α7 helix, which can adopt at least four different conformations dependent on the status of ATP hydrolysis in the C1 domain^14^. Exceptional quenching of W157 fluorescence in the S/pT state (bar *k*, Fig. 3) is likely due to these hydrolysis-coupled transitions.

KaiB-induced fluorescence quenching of KaiC^S157W^ (Fig. 5a) but not KaiC^WT^ (Supplementary Fig. S5) indicates that W157, located on the inner-radius side of the C1 ring, is a sensitive probe for the conformational rearrangements that occur during assembly with KaiB. The crystal structures of the KaiB–KaiC complex from *Thermosynechococcus elongatus* (PDB code: 5JWO & 5JWQ)^15^ show that C_β_ atom of A158 (S157 in *Synechococcus elongatus*) is more than 12 Å away from the closest atom of bound KaiB. Therefore, it is reasonable to suggest that fluorescence quenching induced by the addition of KaiB does not originate from a direct contact between W157 and KaiB.

Formation of the KaiB–KaiC complex is a slow process^5,25–27^. Although this slowness is considered as one of the crucial factors responsible for the slow dynamics of the cyanobacterial circadian clock, the mechanism underlying slow but specific KaiB-KaiC interaction remains unknown. In studies of protein–ligand interaction, it is often debated whether a conformational change of a protein occurs before binding with its ligand (conformational selection)^36^ or is instead induced by ligand binding (induced fit)^37^, and a kinetic approach is often used to distinguish between the two extreme scenarios^38,39^. The dependence of relaxation speed on KaiB concentration (Fig. 6c) exhibited the inverse hyperbolic pattern, a clear sign of selecting a particular conformation of KaiC by KaiB^28^. This is further supported by our observation that exchange of ATP with AMP-PNP abolished the fluorescence decay of KaiC^S157W^-D/E (W157) upon addition of KaiB and made KaiC unable to form a complex with KaiB (Fig. 6d–f). According to the conformational selection mechanism (Supplementary Fig. S7), the time scale of fluorescence decay at the KaiB-saturating concentration should correspond to the time scale of a conformational selection of the C1 ring before KaiB binding. Consistency between the 1/*t*_1/2_ value of ~0.5 h^−1^ (10.5 µM KaiB in Fig. 6c) for KaiC^S157W^-D/E and its ATPase activity (12 ± 1 d^−1^) measured in the absence of KaiB suggests that an event required in prior to KaiB binding is the slow structural change of the C1 ring from pre- into post-hydrolysis states. According to Fig. 5a, the decrease in *FI*_app_ was confirmed as the consequence of KaiB binding only for KaiC^S157W^-D/E and KaiC^S157W^-D/T, even though other phospho-mimicking KaiC^S157W^ mutants retained the ATPase activity. Thus, the selection of the C1-ring conformation by KaiB is gated likely through a coupled conformational change of ATPase and phosphorylation states.

On the other hand, it must be noted that KaiB also undergoes a structural change to form the KaiB–KaiC complex^27^. If the structural change of KaiB occurs concomitantly with KaiC binding (induced fit), the hyperbolic pattern in Fig. 6c will be unaffected because of no probes in KaiB in the present study. If KaiC also selects a particular conformation of KaiB as KaiB does on KaiC, the 1/*t*_1/2_ value will be affected somehow but should decrease still in a hyperbolic manner as long as the formation rate of KaiC-binding-competent state of KaiB is similar to or larger than that of KaiB-binding-competent state of KaiC (see Supplementary Text for details). Because the KaiB-saturating concentration in Fig. 6c is within the range in which robust circadian rhythm can be observed^40^, it is reasonable to propose that the selection of the post-hydrolysis C1-ring by KaiB is so important as that of the KaiB conformation by KaiC as a key mechanism to transmit the slow but stable timing cue from the intra-molecular (KaiC ATPase and KaiB fold-switch) to inter-molecular (KaiB–KaiC binding) scales^14^. In fact, in a recent model of the Kai oscillator^41^, the ATP hydrolysis in the C1 ring is implemented as a key event driving/switching both structural transitions of KaiC and KaiA/KaiB interactions.

In summary, by installing Trp-fluorescence probes at multiple specific sites, we demonstrated that the C1 domain indeed undergoes conformational changes on the inner-radius interface and in the D^145^S^146^ peptide, while maintaining its hexameric ring framework. These C1-ring rearrangements, coupled to ATP hydrolysis events and phosphorylation state, are the origins of the slow circadian period^14^ and provide the basic timing cue for assembly with KaiB^15,16,24,42–44^.

## Materials and Methods

### Expression and purification of Kai proteins

Site-directed mutagenesis was conducted using the QuikChange Mutagenesis Kit (Stratagene). Recombinant Kai proteins were expressed in *E. coli* and purified as described previously^9^.

### Biochemical assays of Kai proteins

All experiments on KaiC^WT^ and its mutants, except for KaiC^S146W^, were carried out in a buffer containing 50 mM Tris, 150 mM NaCl, 5 mM MgCl_2_, 0.5 mM EDTA, and 1 mM ATP at pH 8.0. In the case of KaiC^S146W^, the ATP concentration was increased up to 3 mM to maintain its hexameric conformation stably. KaiC auto-dephosphorylation was initiated by a temperature-jump from an ice bath to 30 °C, and the relative abundances of the phosphorylation states at each time point were analyzed by SDS-PAGE and quantified using *LOUPE*^17^. ATPase measurements were performed by HPLC as described previously^12^.

### Fluorescence spectroscopy

Fluorescence emission spectra from Trp residues were collected every 1 nm with a 0.1 s response time and a scan speed of 1200 nm min^−1^ at an excitation wavelength of 295 nm (Hitachi, F-7000) as described previously^12^. The observed spectra were normalized against both KaiC concentration and the fluorescence signal of an N-acetyl-L-tryptophanamide (NATA) standard solution with an absorbance of 0.05 at 280 nm.

### Data processing and analysis of kinetic measurements

For investigations of the KaiC auto-dephosphorylation process, each sample was stored on ice, transferred to 30 °C, and incubated for 10 min prior to measurements. Time courses were analyzed by using equation (1) (see text). Fitting quality was assessed by the value of residual sum of squares (RSS) between experimental and simulated *FI*s. For studies of kinetics upon addition of KaiB, KaiC was pre-incubated at 30 °C for at least 2 h, and then mixed with KaiB.

### Size exclusion chromatography

KaiB (0.24 mg/ml) and KaiC (1.2 mg/ml) had been pre-incubated separately at 30 °C for 2 h, and then equal volumes of two samples were mixed together (*t* = 0). An aliquot (500 μL) of the mixture was loaded onto a Superdex 200 10/30 GL column (GE Healthcare) immediately after the mixing (*t* = ~2 min), or after further incubation at 30 °C for 18 h. The column was equilibrated in a buffer containing 50 mM Tris, 150 mM NaCl, 5 mM MgCl_2_, 0.5 mM EDTA, 1 mM DTT, and 0.5 mM ATP or AMP-PNP at pH 8.0. Analysis was performed at a flow rate of 0.5 mL/min at room temperature.

### Crystallization and Refinements

Crystallization of KaiC1^S229W^ was conducted as described^14^. Reflections at 50–2.8 Å were collected at 100 K at BL44XU of SPring-8. The initial structure was determined by molecular replacement using the template model 4TL7 (the crystal structure of KaiC1^WT^ solved at 1.93 Å). Crystallographic statistics and refinement parameters are provided in Supplementary Table S1 (see Supplementary Text for details).

### Data availability

The datasets generated and/or analyzed during the current study are available from the corresponding author upon reasonable request.

## Electronic supplementary material

Supplementary Information
