# Author Correction: Conformational rearrangements of the C1 ring in KaiC measure the timing of assembly with KaiB

**DOI:** 10.1038/s41598-020-58954-z

**Published:** 2020-02-11

**Authors:** Atsushi Mukaiyama, Yoshihiko Furuike, Jun Abe, Shin-ichi Koda, Eiki Yamashita, Takao Kondo, Shuji Akiyama

**Affiliations:** 1 Research Center of Integrative Molecular Systems (CIMoS), Institute for Molecular Science, National Institute for Natural Sciences, 38 Nishigo-Naka, Myodaiji, Okazaki 444-8585 Japan; 2 Department of Functional Molecular Science, SOKENDAI (The Graduate University for Advanced Studies), 38 Nishigo-Naka, Myodaiji, Okazaki 444-8585 Japan; 3 Institute for Protein Research, Osaka University, 3-2 Yamada-oka, Suita, 565-0871 Japan; 4 Division of Biological Science, Graduate School of Science, Nagoya University, Furo-cho, Chikusa-ku, Nagoya, 464-8602 Japan

Correction to: *Scientific Reports* 10.1038/s41598-018-27131-8, published online 11 June 2018

Shin-ichi Koda was omitted from the author list in the original version of this Article. This has been corrected in the PDF and HTML versions of the Article, and in the accompanying Supplementary Information file.

The Author Contributions section now reads:

A.M. and S.A. designed the experiments. A.M. conducted biochemical and fluorescence experiments and analyzed the data with inputs from S.K. J.A. screened initial crystallization condition. Y.F., J.A. and S.A. collected the diffraction data and analyzed the structures with inputs from E.Y. Both A.M. and S.A. drafted the manuscript with input from all authors.

