# Supplementary material for: Conformational rearrangements of the C1 ring in KaiC measure the timing of assembly with KaiB
Source: Sci Rep. 2018 Jun 11;8:8803. doi: 10.1038/s41598-018-27131-8 (PMC5995851; doi:10.1038/s41598-018-27131-8)
Supplement: Supplementary file 1 — Supplementary Information [file 41598_2018_27131_MOESM1_ESM.pdf]

Supplementary Information for

Title: Conformational rearrangements of the C1 ring in KaiC measure the timing of assembly with KaiB

Authors: Atsushi Mukaiyama[1,2,*], Yoshihiko Furuike[1,2], Jun Abe[1], Shin-ichi Koda[1,2], Eiki Yamashita[3], Takao Kondo[4] and Shuji Akiyama[1,2,*]

Author Affiliation: [1]Research Center of Integrative Molecular Systems (CIMoS), Institute for Molecular Science, National Institute for Natural Sciences, 38 Nishigo-Naka, Myodaiji, Okazaki 444-8585, Japan. [2]Department of Functional Molecular Science, SOKENDAI (The Graduate University for Advanced Studies), 38 Nishigo-Naka, Myodaiji, Okazaki 444-8585, Japan. [3]Institute for Protein Research, Osaka University, 3-2 Yamada-oka, Suita 565-0871, Japan. [4]Division of Biological Science, Graduate School of Science, Nagoya University, Furo-cho, Chikusa-ku, Nagoya 464-8602, Japan. *To whom correspondence may be addressed. Email: amukai@ims.ac.jp or akiyamas@ims.ac.jp

Contents

Supplementary Method

Data processing of X-ray diffraction data

Simulation of reaction kinetics

Supplementary Figure

Figure S1 Arrhythmia or rhythmic abnormality of KaiC phosphorylation cycles caused by tryptophan mutagenesis

Figure S2 Time-evolutions of the ratio of phosphorylated KaiC in the presence of KaiA and KaiB.

Figure S3 Tryptophan fluorescence intensity (FI_i) for KaiC^{WT}, KaiC^{W92F}, KaiC^{S146W}, KaiC^{S157W}, and KaiC^{S229W} as functions of phosphorylation state (i = S/pT, pS/pT, pS/T, or S/T) and oligomerization state (hexamer or monomer)

Figure S4 Trp-fluorescence spectra of KaiC^{WT}, KaiC^{S146W}, and KaiC^{S157W} in monomeric form

Figure S5 Apparent fluorescence intensity (FI_{app}) of the phospho-mimicking mutants, S/E, D/E, D/T and A/A of KaiC^{WT} (no artificial Trp probes) after 24 h incubation at 30°C with or without an equimolar concentration of KaiB (monomer basis)

Figure S6 Raw images of Figs. 5**c** (**a**) and 6**f** (**b**)

Figure S7 Conformational selection and induced-fit models

Figure S8 Effect of the formation rate (k_{b+}) of KaiC-binding-competent state (B*) of KaiB on the overall rate for the complex formation

Supplementary Table

Table S1 Data collection and refinement statistics

References

Supplementary Text

Data processing of X-ray diffraction data

Data were processed using *HKL2000* [1], and the reflections from 50 to 2.8 Å were merged. The initial structure was determined by the molecular replacement method using *Molrep* [2] using the template model of 4TL7 (the crystal structure of KaiC1^{WT} solved at 1.93 Å). Refinement and model building were performed using *Refmac5* [3] and *COOT* [4]. Crystallographic statistics and refinement parameters are provided in Table S1. Solvent-accessible surface area was calculated using *AreaIMol* in the CCP4 program suite [5], with probe radius set to 1.4 Å.

Simulation of reaction kinetics

We examined possible effects of KaiB on the data in Fig. 6**c** by simulating reaction kinetics of following two schemes. In Scheme I, both KaiB (B) and KaiC (C) selects a particular state upon the complex formation:

$$\mathrm{C} \underset{k_{\mathrm{c-}}}{\overset{k_{\mathrm{c+}}}{\rightleftharpoons}} \mathrm{C}^*$$

$$\mathrm{B} \underset{k_{\mathrm{b-}}}{\overset{k_{\mathrm{b+}}}{\rightleftharpoons}} \mathrm{B}^* \qquad \text{(Scheme I)}$$

$$\mathrm{C}^* + \mathrm{B}^* \underset{k_{\mathrm{off}}}{\overset{k_{\mathrm{on}}}{\rightleftharpoons}} \mathrm{C}^*\mathrm{B}^*$$

where C* and B* represents KaiB-binding-competent and KaiC-binding-competent states, respectively. To simulate our experimental condition, initial concentrations of C, C*, and C*B* were given as $[C_0]$ = 2.985 μM, $[C^*_0]$ = 0.015 μM, and $[C^*B^*_0]$ = 0 μM, respectively. The total initial concentration of KaiC is thus 3 μM ($[C_0^{total}] = [C_0] + [C^*_0] + [C^*B^*_0]$). The minor population of C* before addition of KaiB is supported by the absence of detectable burst phase in the fluorescence change upon addition of KaiB (Fig. 5D). Initial concentrations of B and B* were varied so that a total initial concentration of KaiB ($[B_0^{total}] = [B_0] + [B^*_0]$) before mixing varied from 1 to 9 μM, but while keeping a constant ratio of $[B^*_0]$ to $[B_0^{total}]$ at 0.074 as in the previous report [6]. Rate constants regarding the state change of KaiC (k_{c+} = 0.5 h^{-1} and k_{c-} = 100 h^{-1}) and complex formation (k_{on} = 5×10^9 M^{-1} h^{-1} and k_{off} = 0.01 h^{-1}) were fixed to reasonable values during the simulation.

Figure S8**a** shows a time evolution of concentration profiles simulated under a typical condition, in which the equimolar of KaiB ($[B_0]$ = 2.78 μM, $[B^*_0]$ = 0.22 μM, $[B_0^{total}]$ = 3 μM) is added to KaiC at t = 0 (k_{b+} = 1.16 h^{-1}, and k_{b-} = 14.6 h^{-1}). In this case, both [B] and [C] decreased in the same time scale as [C*B*] increased without accumulating [C*], because the overall reaction is rate-limited by k_{c+}. A $1/t_{1/2}$ value was estimated from a time-course (Fig. S8**b**) of the total concentration of C* ($[C^{*,total}]$ = [C*] + [C*B*]) and then plotted in Fig. S8**c** (blue circles) against $[B_0^{total}]$. A series of simulations using various exchange rates of KaiB (k_{b+} and k_{b-}) reproduced a decrease of the $1/t_{1/2}$ value with increasing KaiB concentration essentially when k_{b+} is larger than k_{c+} (blue circles, green triangles, and gray inversed triangles). This result suggests that the formation rate of the KaiC-binding-competent state of KaiB can influence the curvature of the hyperbolic decrease in Fig. 6**c**, but that both k_{b+} and k_{b-} can be larger than k_{c+} (= 0.5 h^{-1}).

In Scheme II, KaiB is assumed to undergo the state change concomitantly with KaiC binding.

$$\mathrm{C} + \mathrm{B} \underset{k_{c-}}{\overset{k_{c+}}{\rightleftharpoons}} \mathrm{C}^* + \mathrm{B} \underset{k_{\mathrm{off}}}{\overset{k_{\mathrm{on}}}{\rightleftharpoons}} \mathrm{C}^*\mathrm{B} \underset{k_{b-}}{\overset{k_{b+}}{\rightleftharpoons}} \mathrm{C}^*\mathrm{B}^* \qquad \text{(Scheme II)}$$

The $1/t_{1/2}$ value should be saturated hyperbolically with increasing $[B_0^{total}]$ nearly irrespective of k_{b+} as observed in Fig. 6**c**, because our probe installed solely to KaiC is not sensitive enough to distinguish between C*B and C*B*.

Supplementary Figures

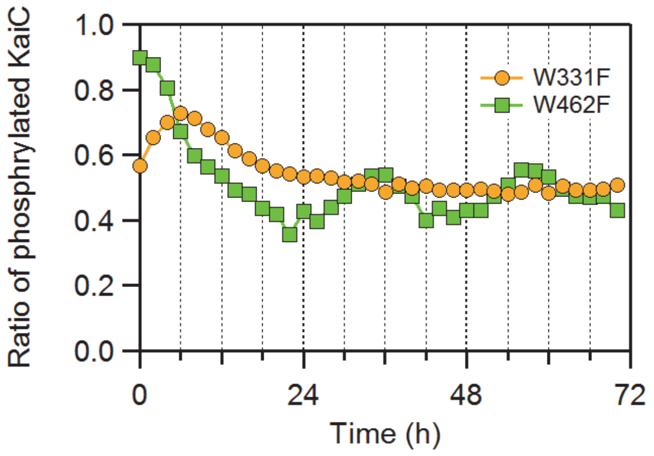

Figure S1. Arrhythmia or rhythmic abnormality of KaiC phosphorylation cycles caused by tryptophan mutagenesis. KaiC mutants were co-incubated with KaiA and KaiB, and then analyzed as previously reported [7,8].

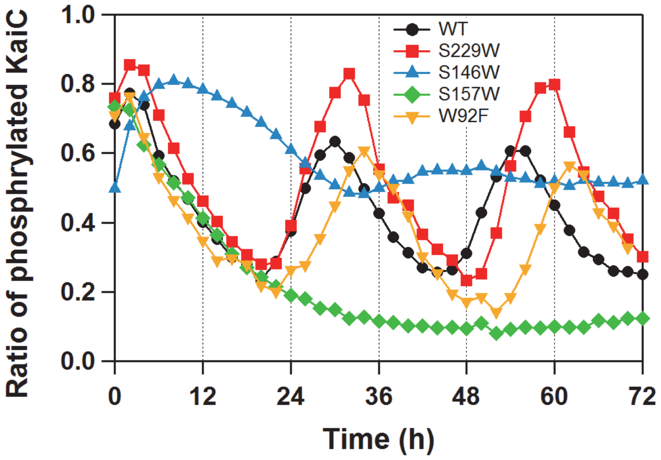

Figure S2. Time-evolutions of the ratio of phosphorylated KaiC in the presence of KaiA and KaiB. $KaiC^{WT}$ or each of mutants was co-incubated with KaiA and KaiB, and then analyzed as previously reported [7,8].

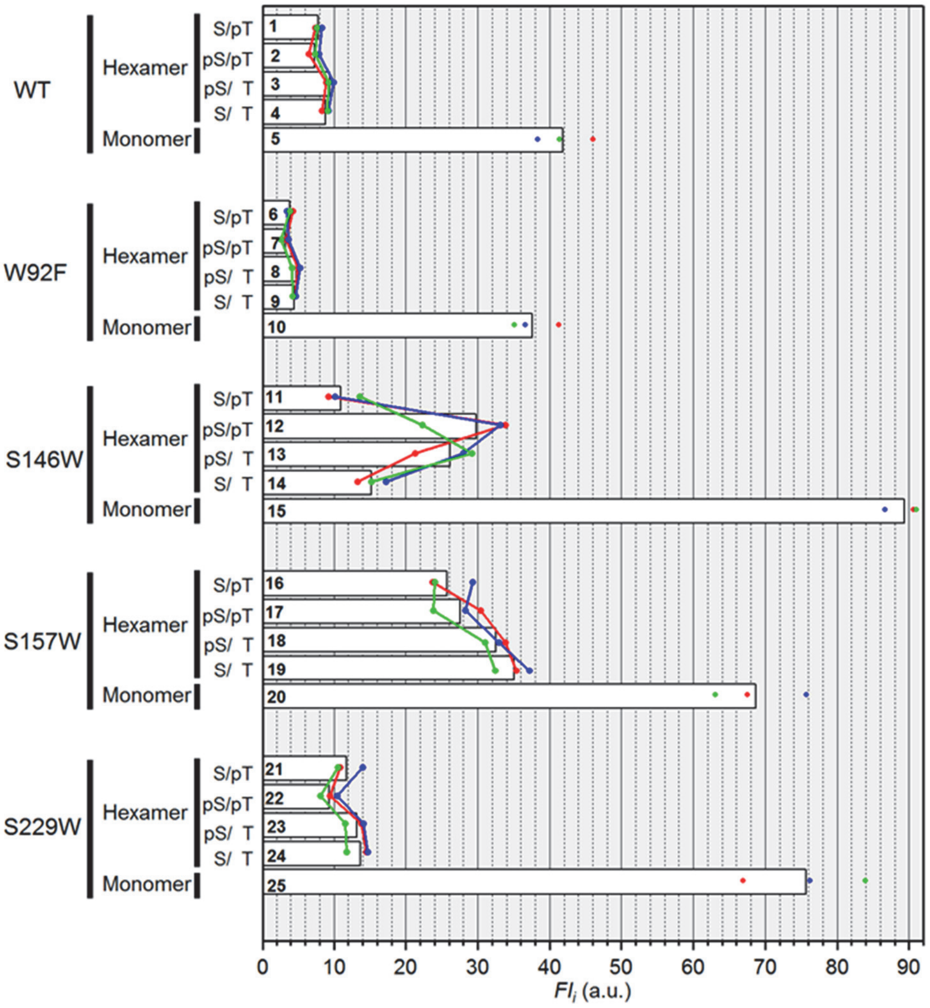

Figure S3. Tryptophan fluorescence intensity (FI_i) for KaiC^{WT}, KaiC^{W92F}, KaiC^{S146W}, KaiC^{S157W}, and KaiC^{S229W} as functions of phosphorylation state (i = S/pT, pS/pT, pS/T, or S/T) and oligomerization state (hexamer or monomer). Each dataset is shown in a different color as filled circles connected with a line, and the mean value from three independent experiments is represented as bars. The *FI* values shown by filled circles for bars *5*, *10*, *15*, *20*, and *25* were estimated from fluorescence spectra (Figs. 4**a**&S4), and those shown by the other were determined by using equation (1) in the main text [9].

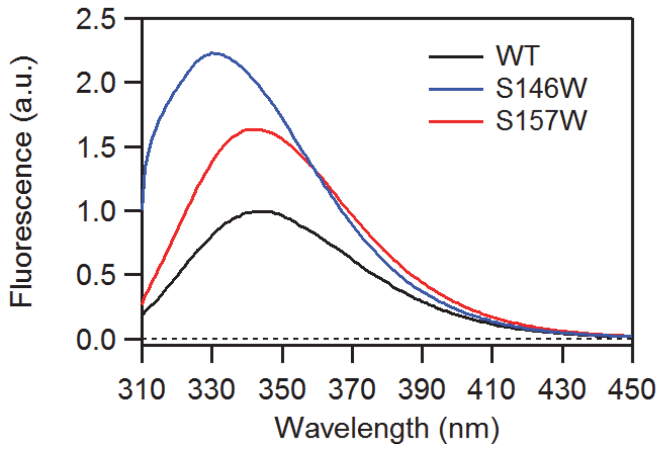

Figure S4. Trp-fluorescence spectra of KaiC^{WT}, KaiC^{S146W}, and KaiC^{S157W} in monomeric form.

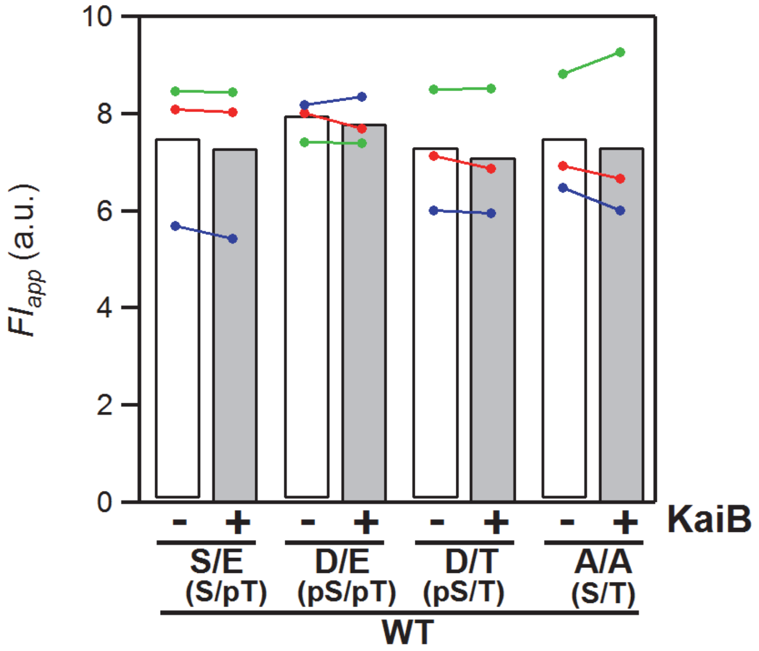

Figure S5 Apparent fluorescence intensity (FI_{app}) of the phospho-mimicking mutants, S/E, D/E, D/T and A/A of KaiC^{WT} (no artificial Trp probes) after 24 h incubation at 30°C with or without an equimolar concentration of KaiB (monomer basis). Each dataset is shown in a different color as a pair of filled circle connected with a line, and the mean value from independent measurements is represented as bars.

a

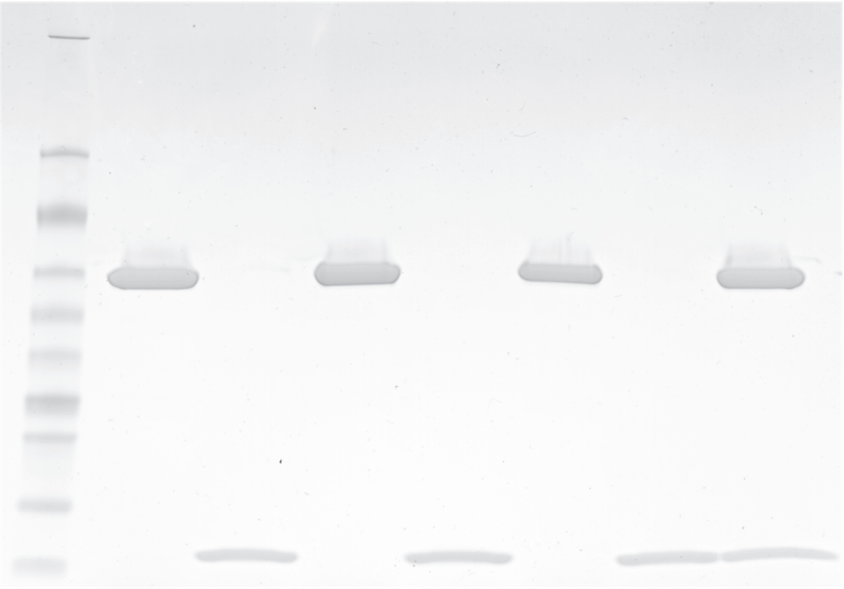

b

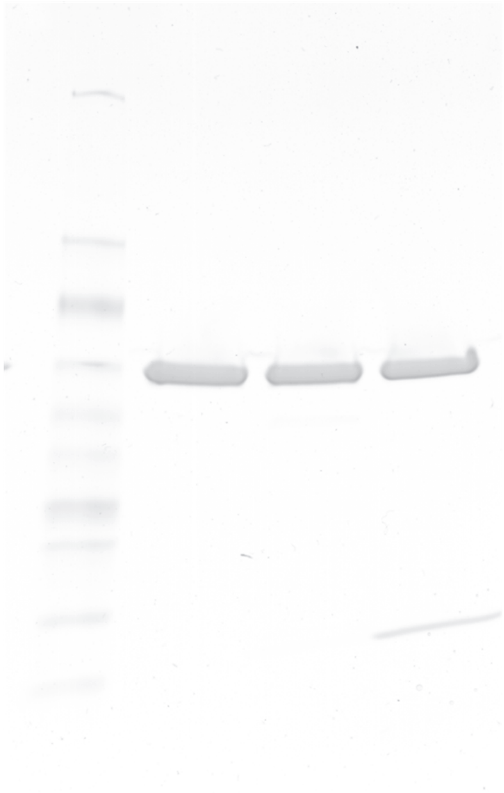

Figure S6 Raw images of Figs. 5**c** (**a**) and 6**f** (**b**)

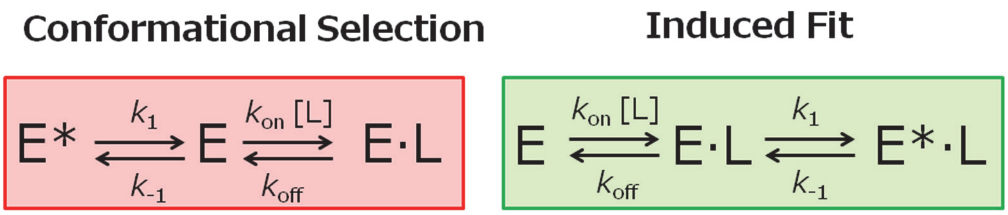

E : Enzyme L : Ligand

Rapid equilibrium approximation ($k_{off} + k_{on}[L] >> k_1 + k_{-1}$)

$$k_{obs} = k_1 + k_{-1}\frac{K_d}{K_d + [L]} \qquad k_{obs} = k_{-1} + k_1\frac{[L]}{K_d + [L]}$$

$$K_d = k_{off} / k_{on}$$

$[L] = 0 \quad k_{obs} = k_1 + k_{-1}$ $\qquad$ $[L] = 0 \quad k_{obs} = k_{-1}$

$[L] = \infty \quad k_{obs} = k_1$ $\qquad$ $[L] = \infty \quad k_{obs} = k_1 + k_{-1}$

Figure S7. Conformational selection and induced-fit models.

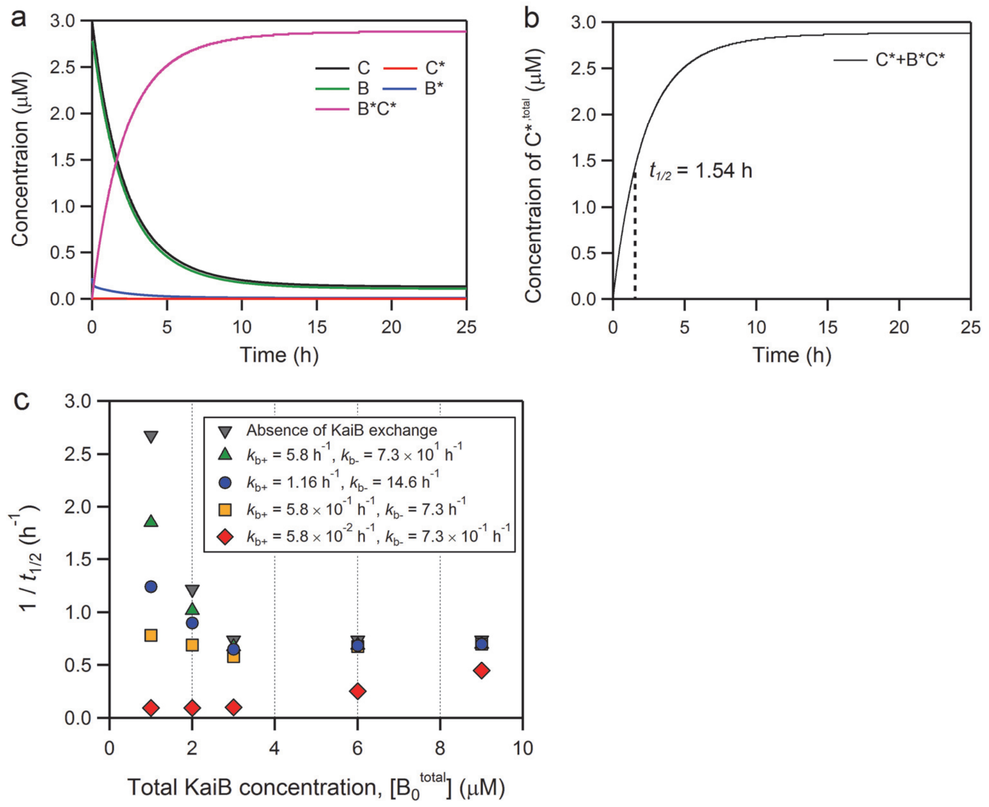

Figure S8. Effect of the formation rate (k_{b+}) of KaiC-binding-competent state (B*) of KaiB on the overall rate for the complex formation. See details in Supplementary Text. (**a**) Simulated time-courses of concentration profiles after mixing KaiC ($[C_0]$ = 2.985 μM, $[C^*_0]$ = 0.015 μM, $[C^*B^*_0]$ = 0 μM, and $[C_0^{total}]$ = 3 μM) with equimolar of KaiB ($[B_0]$ = 2.78 μM, $[B^*_0]$ = 0.22 μM, $[B_0^{total}]$ = 3 μM) using rate constants of k_{c+} = 0.5 h^{-1}, k_{c-} = 100 h^{-1}, k_{b+} = 1.16 h^{-1}, k_{b-} = 14.6 h^{-1}, k_{on} = 5×10^9 M^{-1} h^{-1}, and k_{off} = 0.01 h^{-1}. (**b**) Time evolution of the total concentration of KaiB-binding-competent state of KaiC ($[C^{*,total}]$ = $[C^*]$ + $[C^*B^*]$). $[C^{*,total}]$ was reproduced by using the data shown in Fig. S8**a**. (**c**) Dependence of $1/t_{1/2}$ on the total KaiB concentration ($[B_0^{total}]$). Each reaction rate for k_{c+}, k_{c-}, k_{on}, and k_{off} was fixed to the value used in Fig. S8**b**, but the formation rate of KaiC-binding-competent state (k_{b+}) was varied while keeping a constant ratio between k_{b+} and k_{b-}.

Supplementary Table

Table S1. Data collection and refinement statistics

Protein	KaiC1^{S229W}
Space group	$P3_121$
Unit cell size / Å	*a*=108.1, *c*=224.1
Resolution range / Å	50–2.8 (2.9–2.8)
Number of all reflections	408014
Number of unique reflections	37912 (3743)
Redundancy	10.8 (10.8)
Completeness (%)	100 (100)
R_{merge}	8.6 (>100)
<*I*>/sigma<*I*>	20.3 (2.4)
R_{work} (%) [a]	24.1
R_{free} (%) [a]	30.0
RMS bond length / Å	0.007
RMS bond angle (°)	1.26
Ramachandran favored (%)	95.4
Ramachandran allowed (%)	4.0
Cruickshank’s DPI / Å	0.45
PDB code	5YZ8

Values in parentheses correspond to the highest-resolution shell. [a]$R_{work,\ free} = \Sigma|F_{obs}-F_{calc}|/\Sigma F_{obs}$, where the crystallographic R-factor was calculated including and excluding refinement reflections. In each refinement, free reflections constituted 5% of the total number of reflections.

References

1. Otwinowski, Z. & Minor, W. Processing of X-ray diffraction data collected in oscillation mode. *Methods Enzymol.* **276**, 307-326 (1997).
2. Vagin, A. & Teplyakov, A. MOLREP: an Automated Program for Molecular Replacement. *Journal of Applied Crystallography* **30**, 1022-1025 (1997).
3. Murshudov, G. N., Vagin, A. A. & Dodson, E. J. Refinement of Macromolecular Structures by the Maximum-Likelihood Method. *Acta Crystallogr. D, Biol. Crystallogr.* **53**, 240-255 (1997).
4. Emsley, P. & Cowtan, K. Coot: model-building tools for molecular graphics. *Acta Crystallogr. D, Biol. Crystallogr.* **60**, 2126-2132 (2004).
5. Winn, M. D. *et al.* Overview of the CCP4 suite and current developments. *Acta Crystallogr. D, Biol. Crystallogr.* **67**, 235-242 (2011).
6. Chang, Y. G. *et al.* A protein fold switch joins the circadian oscillator to clock output in cyanobacteria. *Science* **349**, 324-328 (2015).
7. Nakajima, M. *et al.* Reconstitution of circadian oscillation of cyanobacterial KaiC phosphorylation in vitro. *Science* **308**, 414-415 (2005).
8. Nishiwaki, T. *et al.* A sequential program of dual phosphorylation of KaiC as a basis for circadian rhythm in cyanobacteria. *EMBO J.* **26**, 4029-4037 (2007).
9. Murayama, Y. *et al.* Tracking and visualizing the circadian ticking of the cyanobacterial clock protein KaiC in solution. *EMBO J.* **30**, 68-78 (2011).
